# Marked decrease in serum pepsinogen II levels resulting from endoscopic resection of a large duodenal tumor

**DOI:** 10.1007/s12328-014-0534-y

**Published:** 2014-11-07

**Authors:** Tomoyuki Yada, Koichi Ito, Keigo Suzuki, Koki Okubo, Yoichiro Aoki, Naoki Akazawa, Hitohiko Koizuka, Tsuyoshi Ishida, Naomi Uemura

**Affiliations:** 1 Department of Gastroenterology, Kohnodai Hospital, National Center for Global Health and Medicine, 1-7-1 Kohnodai, Ichikawa, Chiba 272-8516 Japan; 2 Department of Pathology and Laboratory Medicine, Kohnodai Hospital, National Center for Global Health and Medicine, 1-7-1 Kohnodai, Ichikawa, Chiba 272-8516 Japan

**Keywords:** Pepsinogens, Duodenal neoplasms, Endoscopic resection

## Abstract

Studies have indicated that serum pepsinogen (PG) levels are not only markers for chronic atrophic gastritis but also predictive risk factors for gastric cancer. However, serum PG levels can change because of pathological conditions other than gastritis. We report the first case in which abnormally high serum PG II levels (168.8 ng/mL) led to the discovery of a large tumor covering a wide area in the duodenum, and after resection of the tumor, the serum PG II levels markedly decreased. Because endoscopic and histopathological examinations showed no indications of atrophic changes, inflammation of the gastric mucosa, or *Helicobacter pylori* infection, the serum PG II levels eventually returned to normal (10.1 ng/mL). The preoperative abnormally high PG II levels were probably caused by the large duodenal tumor that prevented PG II (which is produced by the duodenal Brunner’s glands) from being secreted into the lumen, a condition that increased the amount transferred to the bloodstream. No previous reports have investigated serum PG II levels before and after resection of a large duodenal tumor. We believe this case provides valuable insight regarding the dynamics of PG II in the body and has important diagnostic implications.

## Introduction

Pepsinogen (PG) is a simple, noninvasive, and effective serum marker for monitoring the progression of chronic atrophic gastritis (CAG) [1, 2]. PG is an inactive precursor of pepsin, a proteolytic enzyme in gastric juice, and exists in two forms—PG I and PG II [3]. PG I is secreted by the fundic glands, which are primarily involved in gastric acid secretion. PG II is secreted not only by the fundic glands but also by the cardiac, pyloric, and duodenal Brunner’s glands. Approximately 99 % of PG is secreted into the gastroduodenal lumen, and the remaining 1 % enters the bloodstream. Gastric mucosal atrophy is accompanied by early decreases in serum PG I levels, but serum PG II levels remain relatively stable. The serum PG I level and the ratio of serum PG I to PG II decrease in stages, which reflects the degree of gastric mucosal atrophy. Therefore, these serum levels can be used as indicators of the progression of gastric mucosal atrophy in patients with CAG. Previous studies have indicated that because the risk of gastric cancer increases with gastric mucosal atrophy in patients with CAG [4], these PG levels are useful for assessing the risk of gastric cancer in screening tests (PG method) [5]. In Japan, the PG method and measurement of serum anti-*Helicobacter pylori* (*Hp*) antibody are concurrently used to identify patients at high risk for gastric cancer [6, 7]. These tests are both simple and effective; however, it should be noted that pathological conditions other than gastritis, such as that caused by *Hp* infection, can cause abnormalities in serum PG levels. In this study, we report a case of a patient who showed a marked decrease in elevated serum PG II levels after endoscopic resection of a large duodenal tumor, which was identified by abnormally high serum PG II levels.

## Case report

**Fig. 1:**
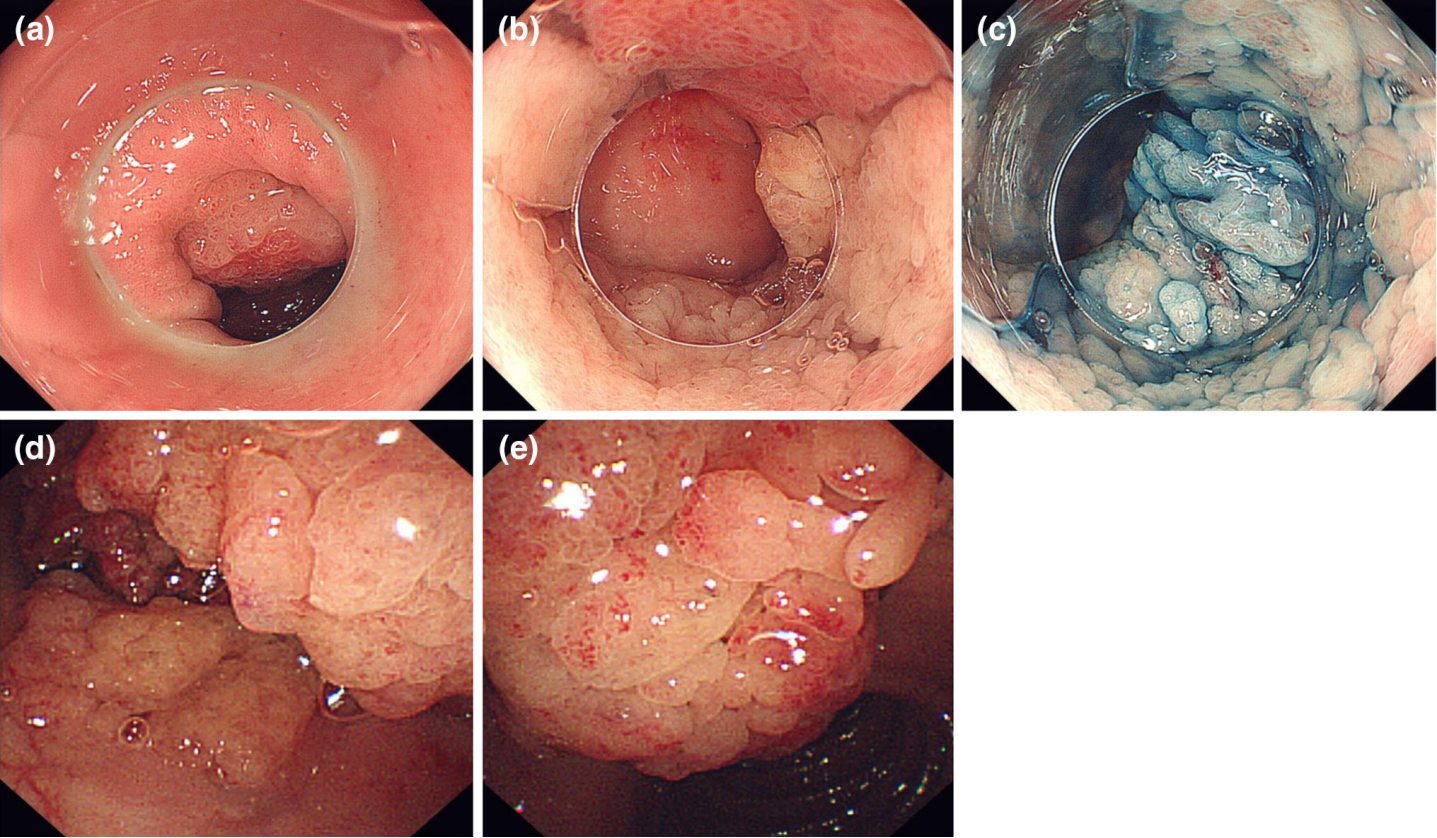
At the time of the initial endoscopic examination, we identified a 60-mm 0–IIa + I type lesion extending from the duodenal bulb to the second part of the duodenum. **a–c** Subcircumferential flat elevated lesions in the duodenal bulb are shown. **d**, **e** The tall protrusion is continuous from the anal side of the flat elevated lesion in the bulb to the second portion (observation using a side-view endoscope)

**Fig. 2:**
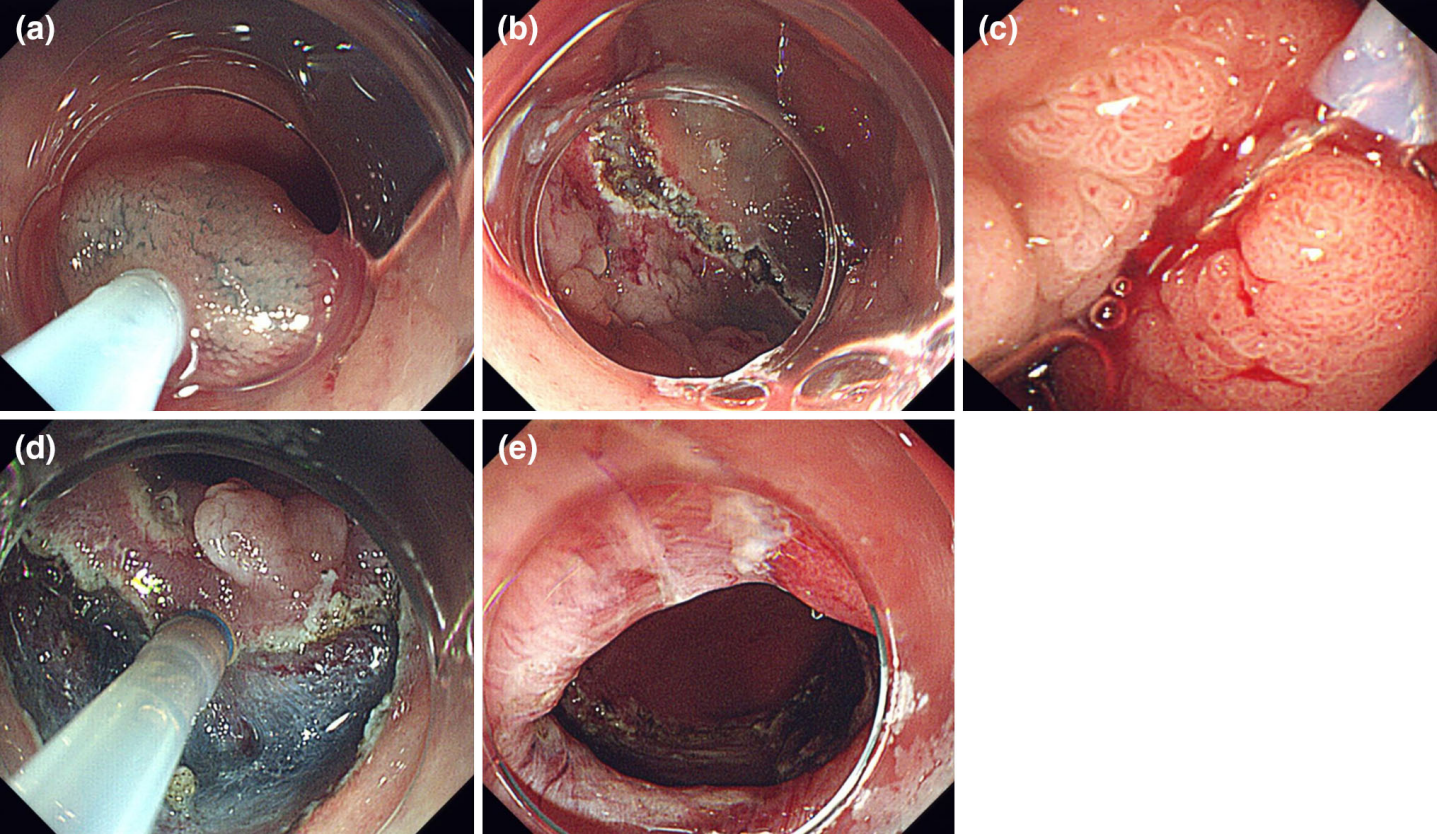
During treatment **a** local injection, **b** mucosal cutting around the flat elevated lesion, **c** resection of the 0–I area using a snare and a side-view endoscope, **d** continuous dissection, **e** post-treatment ulcer

**Fig. 3:**
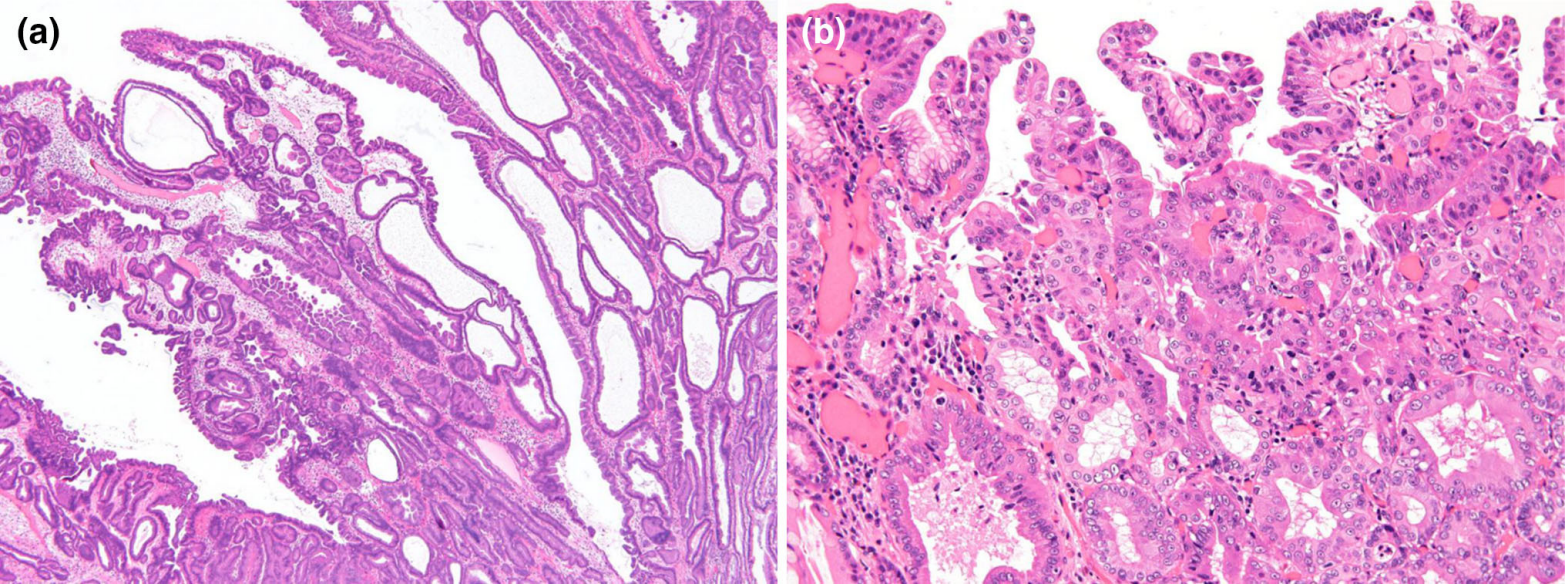
Histopathological findings of the duodenal tumor. **a** Low-power view. **b** High-power view

The patient was an asymptomatic 65-year-old female. Blood tests performed as part of a gastric cancer screening program run by the patient’s local government revealed abnormally high serum PG II levels, so she was referred to our department for further evaluation of upper gastrointestinal (GI) tests. Blood tests at the time of her medical examination were serum PG I, 59.6 ng/mL; serum PG II, 168.8 ng/mL; PG I/II ratio, 0.4 (PG method: positive); and *Hp* antibody, negative (3.0 U/mL). A commercial CLEIA kit (Lumipulse Presto pepsinogen I/II kit; Fujirebio Inc., Tokyo, Japan) was used to measure the serum PG level, and a commercial EIA kit (E-Plate Eiken *H. pylori* antibody; Eiken Chemical Co., Ltd., Tokyo, Japan) was used to measure serum *Hp* antibody levels. Her medical history included hypertension, dyslipidemia, and type 2 diabetes, which were being treated with medications. Routine blood test results were almost normal, and the patient was not taking any acid-suppressing drugs. Upper gastrointestinal (GI) endoscopy performed at our department indicated a subcircumferential villous flat elevated lesion over a wide area in the duodenal bulb (Fig. 1a–c). The anal side of the flat lesion was attached to a long protruding lesion that extended to the second part of the duodenum. The scope could not be reversed within the duodenal bulb because of the protruding lesion, but the entire area could be observed by using a side-view endoscope (Fig. 1d, e). The 60-mm lesion presented as a 0–IIa + I type, and biopsy revealed a tubular adenoma. The surrounding gastric mucosa showed no signs of atrophic change, and the biopsy results were negative for *Hp*. After obtaining informed consent and determining that the patient preferred endoscopic treatment over open surgery, we performed endoscopic resection of the lesions (Fig. 2). First, to define the area of resection, we performed a circumferential mucosal incision in the region of the duodenal bulb where the 0–IIa lesion was located. Then, using a side-view endoscope, we resected the area where the 0–I lesion was located with a snare, after which it became possible to reverse the scope within the duodenal bulb. We were thus able to perform piecemeal resection of the lesion via endoscopic submucosal dissection and endoscopic mucosal resection. Histopathological analysis revealed that the lesion was a well-differentiated papillotubular adenocarcinoma, with tubulovillous adenoma confined within the mucosa and no lymphatic and venous invasion (Fig. 3a, b). Given that the resection was performed over a wide area, we performed endoscopic balloon dilatation twice as a preventative measure against stenosis.

**Fig. 4:**
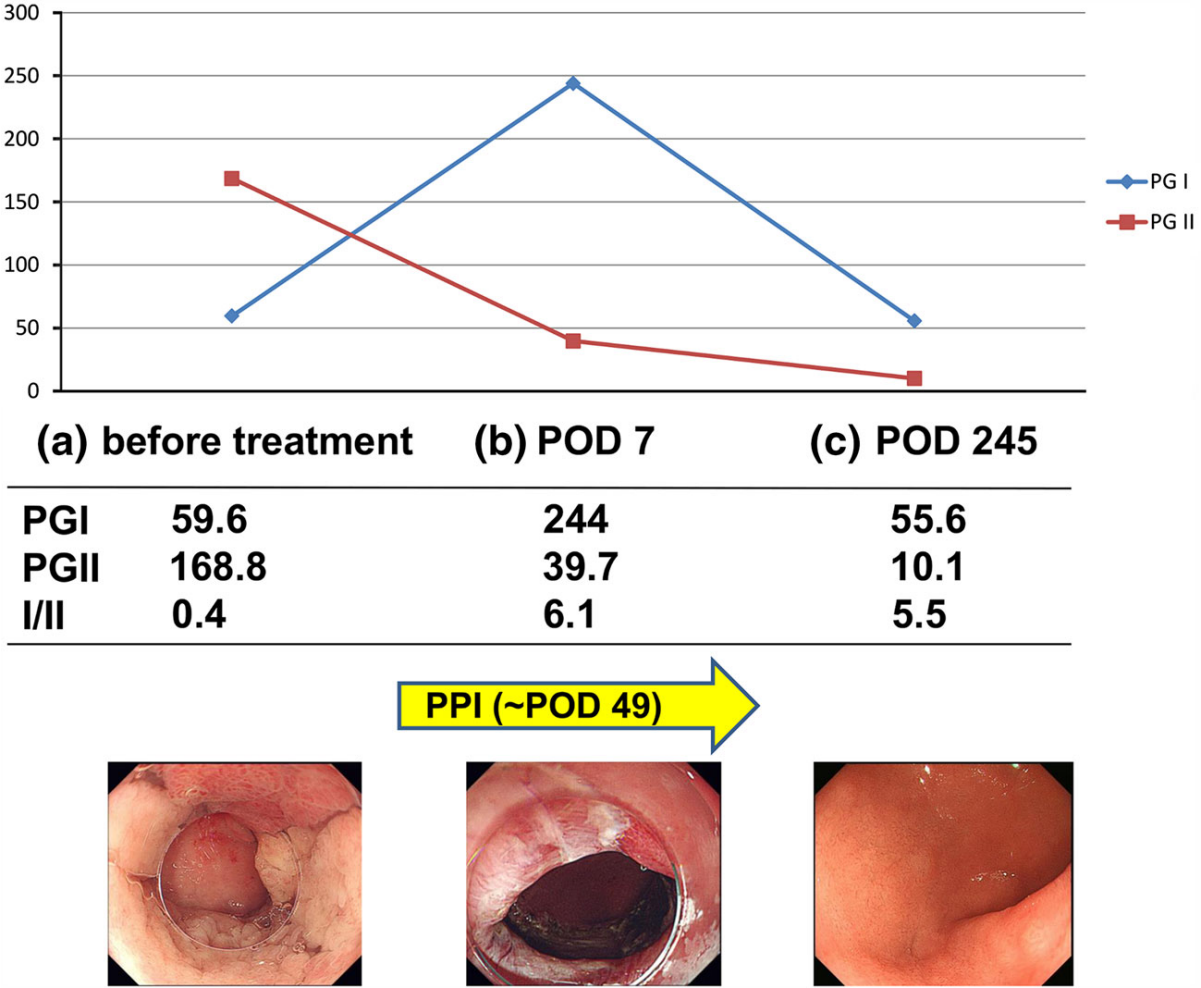
Changes in the clinical course and serum PG levels. **a** Before endoscopic treatment. **b** After endoscopic treatment and during proton-pump inhibitor (PPI) administration. **c** Cicatrization after stopping PPI administration

We administered a proton-pump inhibitor (PPI) from the day of surgery. On postoperative day (POD) 49, we confirmed scar formation without signs of stenosis and then stopped PPI administration. The serum PG I and II levels on POD 7 were 244 and 39.7 ng/mL, respectively, and on POD 245, they were 55.6 and 10.1 ng/mL, respectively, which indicated a marked decrease in serum PG II levels due to tumor resection (Fig. 4). At 8-month follow-up, there were no signs of cancer recurrence.

## Discussion

To the best of our knowledge, this is the first report to describe variations in serum PG II levels before and after resection of a duodenal tumor.

The serum PG level was originally used as a serum marker to measure the degree of CAG [1, 2]. However, because studies have established a correlation between atrophic gastritis and gastric cancer [4], progression of CAG, which often develops into gastric cancer, has been assessed on the basis of decreases in serum PG I levels and the serum PG I/II ratio, thereby affording an effective means of screening for gastric cancer [5]. The reference values most often used are PG I levels of ≤70 ng/mL and a PG I/II ratio of ≤3.0 [1]. Some reports differ with respect to the accuracy of this method for detection of cancer; however, meta-analysis has established a sensitivity of 77.3 % and a specificity of 73.2 % [8].

Studies have shown that risk assessment using a combination of the PG method and *Hp* antibody measurements is effective [9, 10], and consequently, the number of municipalities in Japan that employ this method in medical examinations is increasing. Watabe et al. [9] used the combination of the PG method and *Hp* antibody measurement to investigate the incidence rate of gastric cancer in the following four groups—group A (PG method, negative; *Hp* antibody, negative), group B (PG method, negative; *Hp* antibody, positive), group C (PG method, positive; *Hp* antibody, positive), and group D (PG method, positive; *Hp* antibody, negative). In general, patients in group A were not infected with *Hp*, patients in group B were infected with *Hp* but presented with only slight atrophy, patients in group C were infected with *Hp* and presented with advanced atrophy, and patients in group D presented with severe intestinal metaplasia due to advanced CAG, a condition in which it was determined that *Hp* was naturally killed. On the basis of these results, it was determined that the risk for gastric cancer progressively increased from group A, in which the risk was extremely low, to groups B, C, and D, in which the risk was highest.

On the basis of the above classification, our patient was placed in group D. However, as mentioned above, because patients in group D generally present with highly advanced atrophic gastritis, serum PG II levels are usually low. In addition, all tests performed on this patient indicated no *Hp* infection. Such patients often present with serum PG II levels of ≤15 ng/mL. The high serum PG II level (168.8 ng/mL) presented by our patient was, therefore, atypical.

In general, abnormal serum PG levels can be caused by a variety of pathological conditions, including renal failure, acute gastric mucosal lesion (AGML), gastroduodenal ulcer, and ingestion of acid-suppressing drugs (especially PPIs) [11–13]. Because PG is excreted by the kidneys, renal failure patients present with high serum PG levels; however, the blood and urine test results of our patient at the initial examination indicated normal renal function. It is believed that AGMLs and peptic ulcers cause cell destruction via severe inflammation of the gastric mucosa, which results in the accumulation of large quantities of PG I and II in the blood. However, at the initial endoscopic examination of our patient, no ulcerated lesions were found in the upper GI tract, a biopsy of the surrounding gastric mucosa revealed no signs of active inflammation, and the patient was negative for *Hp* infection. In addition, although both serum PG I and II levels increase after ingestion of acid-suppressing drugs, our patient had not taken acid-suppressing drugs during the previous 3 months. The PG levels were tested 7 days after tumor resection. Although the serum PG I levels were elevated because of ingestion of PPIs, the serum PG II levels were markedly reduced. On POD 245, which was 6 months after the patient had stopped PPI ingestion, the serum PG I levels had normalized, and the serum PG II levels had further decreased.

Because PG staining is a special type of staining, we were unfortunately unable to perform it on the duodenal tumor tissue in the present case. However, we believe it is unlikely that serum PG II levels were elevated because of a PG II-producing tumor for the following reasons. First, although this phenomenon has not been described in duodenal tumors, past studies regarding the association between the results of PG II staining of cancerous tissue and serum PG II levels in gastric cancer have yielded controversial results [14, 15]. Even in the reports from studies that have shown a correlation between PG II staining of cancerous tissue and serum PG II levels in gastric cancer, no patients with markedly high serum PG II levels, as seen in the present patient, were described. Second, although PG II is produced from inside the mucosa within the stomach, it is produced from Brunner’s glands located in the submucosa in the duodenum. Thus, we believe it to be unlikely that duodenal tumors confined within the mucosa, such as the one in the present patient, produce PG II.

From this clinical course, it was thought that the abnormally high serum PG II levels identified at the initial medical examination could have been a consequence of the large duodenal tumor. The tumor may have disrupted secretion of PG II, which is produced by the duodenal Brunner’s glands and secreted into the duodenal lumen. Normally, about 99 % of PG II is secreted into the duodenal lumen, with only 1 % entering the bloodstream. After tumor resection, secretion of PG II into the lumen normalized, which then could have decreased serum PG II to normal levels. To the best of our knowledge, no report has addressed variations in serum PG II levels after resection of a duodenal tumor. We believe that the proposed mechanism in this case for the increased serum PG II level in a patient with highly advanced atrophic gastritis and no evidence of *Hp* infection, i.e., a patient who should have had a low PG II serum level under these conditions, provides new insight in the study of PG II dynamics in the body and has a clinical diagnostic implication.

In conclusion, we described a case in which abnormally high serum PG II levels led to the discovery of a large tumor covering a wide area in the duodenum. After resection of the tumor, the serum PG II levels decreased. Our findings indicate that a large duodenal tumor can cause abnormally high serum PG II levels, an observation that should be kept in mind when differential diagnoses are considered.

## References

[1] Miki K, Ichinose M, Shimizu A (1987). Serum pepsinogens as a screening test of extensive chronic gastritis. Gastroenterol Jpn.

[2] Kiyohira K, Yoshihara M, Ito M (2003). Serum pepsinogen concentration as a marker of *Helicobacter pylori* infection and the histologic grade of gastritis; evaluation of gastric mucosa by serum pepsinogen levels. J Gastroenterol.

[3] Gritti I, Banfi G, Roi GS (2000). Pepsinogens: physiology, pharmacology pathophysiology and exercise. Pharmacol Res.

[4] Uemura N, Okamoto S, Yamamoto S (2001). *Helicobacter pylori* infection and the development of gastric cancer. N Engl J Med.

[5] Yoshihara M, Hiyama T, Yoshida S (2007). Reduction in gastric cancer mortality by screening based on serum pepsinogen concentration: a case-control study. Scand J Gastroenterol.

[6] Inoue K, Fujisawa T, Haruma K (2010). Assessment of degree of health of the stomach by concomitant measurement of serum pepsinogen and serum *Helicobacter pylori* antibodies. Int J Biol Mark.

[7] Ohata H, Kitauchi S, Yoshimura N (2004). Progression of chronic atrophic gastritis associated with *Helicobacter pylori* infection increases risk of gastric cancer. Int J Cancer.

[8] Dinis-Ribeiro M, Yamaki G, Miki K (2004). Meta-analysis on the validity of pepsinogen test for gastric carcinoma, dysplasia or chronic atrophic gastritis screening. J Med Screen.

[9] Watabe H, Mitsushima T, Yamaji Y (2005). Predicting the development of gastric cancer from combining *Helicobacter pylori* antibodies and serum pepsinogen status: a prospective endoscopic cohort study. Gut.

[10] Mizuno S, Miki I, Ishida T (2010). Prescreening of a high-risk group for gastric cancer by serologically determined *Helicobacter pylori* infection and atrophic gastritis. Dig Dis Sci.

[11] Nakahama H, Tanaka Y, Shirai D (1995). Elevated serum pepsinogens in chronic renal failure patients. Nephron.

[12] Wu MS, Wang HP, Wang JT (1995). Serum pepsinogen I and pepsinogen II, and the ratio of pepsinogen I/pepsinogen II in peptic ulcer diseases: with special emphasis on the influence of the location of the ulcer crater. J Gastroenterol Hepatol.

[13] Agréus L, Storskrubb T, Aro P (2009). Clinical use of proton-pump inhibitors but not H2-blockers or antacid/alginates raises the serum levels of amidated gastrin-17, pepsinogen I and pepsinogen II in a random adult population. Scand J Gastroenterol.

[14] Stemmermann GN, Samloff IM, Hayashi T (1985). Pepsinogens I and II in carcinoma of the stomach: an immunohistochemical study. Appl Pathol.

[15] Kodoi A, Haruma K, Yoshihara M (1993). A clinical study of pepsinogen I and II producing gastric carcinomas. (Article in Japanese) Nihon Shokakibyo Gakkai Zasshi.

